# Editorial: Multidisciplinary critical care medicine – Getting things done across specialties

**DOI:** 10.3389/fmed.2023.1135003

**Published:** 2023-01-18

**Authors:** Peter Korsten, Björn Tampe

**Affiliations:** Department of Nephrology and Rheumatology, University Medical Center Göttingen, Göttingen, Germany

**Keywords:** critical care medicine, multidisciplinary, internal medicine, neurology, biomarkers

## Introduction

Critical Care Medicine is a team sport and requires collaboration not only between different healthcare professionals but also between different specialties. While specialization and sub-specialization are common and needed in modern medicine due to the increasing knowledge and complexity of care, solid knowledge is fundamental for optimal patient care. This becomes most evident in critical care medicine, where patients' lives are at stake, and management decisions are crucial for patient survival. Critical care medicine and its practice differ around the globe. In some countries, anesthesiologists are the leading providers, while in others, there may be dedicated intensivists or chest physicians responsible for the organization of the intensive care unit (ICU). In academic centers, many departments run their ICUs: These may be internists (nephrologists, cardiologists), cardiovascular or abdominal surgeons, neurologists, or anesthesiologists. They all have particular expertise and view on patients and their critical illnesses. With the emerging COVID-19 pandemic and hyperinflammatory syndromes, immunological treatments have gained attractiveness in critical care. In this regard, solid knowledge of new immunological drugs is emerging. With this Research Topic entitled “*Multidisciplinary Critical Care Medicine – Getting Things Done Across Specialties*,” we aimed to gather insights from many (sub-) specialties and invited authors from various specialties to contribute their data and opinions. With this editorial, we will give an overview of the topics covered.

The Research Topic has received many submissions, of which 14 were finally accepted for publication. Of these, 10 were original papers dealing with various aspects of critical care medicine, and four were reviews or opinion papers.

## External and internal influencing factors

In the first paper, Álvarez and Parada share their opinion on pain as an essential yet under-investigated contributing factor for the development of delirium in critically ill patients. They highlight that currently used delirium assessments, such as the Confusion Assessment Method for the intensive care unit (CAM-ICU) (1), do not incorporate pain. While there are indicators that pain may influence attention, further research is required to corroborate this hypothesis.

The following paper, by Naef et al. investigated methods for measuring sound and noise levels in an ICU. In short, they first provided a framework for measuring sound pressure levels and sound sources of two beds in a four-bedroom with four sound level meters and four different observers. Then, they tested the method's feasibility in a concrete ICU setting. They found that their proposed method over 24 h was applicable with a good or very good interrater reliability in most of the assessed domains. The paper provides a basis for future studies in the same field with a clearly defined methodology.

The third paper assessed the impact of oxygen saturation on mortality in mechanically ventilated patients Here, Li et al. provided data from a retrospective analysis of roughly 25,000 patients in China. They analyzed the impact of different oxygen saturations in obese and non-obese using a multivariable regression model. Specifically, they found that oxygen saturation levels of 99–100% were associated with higher mortality in obese patients, whereas lower values (between 89 and 93%) had an increased mortality in non-obese patients. Thus, different target oxygen saturation levels may be applied depending on body weight.

The fourth paper investigating assessments and other factors in ICU patients reported a one-day point prevalence study of implementing the ABCDEF bundle during the COVID-19 pandemic (Liu et al.). The ABCDEF bundle is an evidence-based guide for the optimization of ICU care considering various aspects (2): (A) represents “Assess, Prevent, and Manage Pain,” (B) “Both Spontaneous Awakening Trials (SAT) and Spontaneous Breathing Trials (SBT),” (C) stands for “Choice of analgesia and sedation,” (D) relates to “Delirium: Assess, Prevent, and Manage,” (E) “Early mobility and Exercise,” and (F) represents the concept of “Family engagement and empowerment.” In this paper, Liu et al. investigated the implementation in COVID-19 and non-COVID patients. They found that, at the time of the investigation, implementation of the entire bundle was 0% for non-COVID-19 patients and 1% for COVID-19 patients. The highest implementation rates were reported for the “A” component (64% non-COVID-19, 55% COVID-19 patients), “C” (45 and 61%, respectively), and “D” (39 and 35%, respectively). On the other hand, breathing trials (component “B”), exercise programs (component “E”), and family engagement (component “F”) were implemented, ranging from 10 to 30% of patients.

## Medications and infusions

The next set of papers dealt with medications or infusions and their influence on outcomes or their clinical applications in ICU patients. The first paper was a retrospective study on adverse reactions to intravenous immunoglobulin (IVIg) infusions by Kato et al.. Their study of roughly 750 patients identified female sex, neuromuscular diseases, and higher cumulative IVIg doses (around 10 g/kg of body weight) as risk factors for adverse reactions. Overall, the incidence of adverse events was low, ranging from 1 to 8% for most risk factors. Exceptions to this were neuromuscular diseases (e.g., chronic inflammatory demyelinating polyneuropathy, Guillain-Barre syndrome, myasthenia gravis) with an incidence of about 26% and eosinophilic granulomatosis with polyangiitis with an incidence of almost 19%.

Another study presented by Tseng et al. investigated a long-debated topic in ICU patients, whether lactated ringer's (LR) solution or saline (normal saline, NS) were associated with better or worse outcomes. In their prospective study of 938 patients, the LR group had an overall lower mortality than the NS group (adjusted hazard ratio: 0.59; 95% CI 0.43–0.81). Also, the length of the hospital stay was shorter in the LR group. The differences were more pronounced in patients with chronic pulmonary disease than those with chronic kidney or liver disease. The underlying reasons are not clear yet. However, comorbidities should probably be considered when choosing the type of fluid. A recent systematic review with meta-analysis provided evidence from randomized controlled clinical trials that balanced crystalline solutions may offer mortality benefits in unselected ICU patients (3).

Next, Cao et al. provided a literature review on the use of hemoglobin-based oxygen carrier-201 (HBOC-201), a potential blood substitute that is currently very infrequently used in clinical practice. The authors comprehensively review the available evidence from clinical trials, including surgical and medical conditions. As such, HBOC-201 may have an emerging role as a blood substitute when there is a shortage of blood products. Nevertheless, potential side effects on the cardiovascular system, methemoglobinemia, and liver enzyme abnormalities, among others, must be monitored carefully.

## Biomarkers

The only study included in this Research Topic that investigated a potential new biomarker sought to determine the role of sphingosine-1-phosphate (S1P) in cardiac surgery patients (Greiwe et al.). Sphingosine-1-phosphate is known to have a possible beneficial effect on inflammation, and agonists of S1P have been approved for use in multiple sclerosis (MS) or inflammatory bowel disease (4). In addition, S1P is being investigated In rheumatic diseases, such as rheumatoid arthritis, systemic lupus erythematosus, or systemic sclerosis (4). In their study, Greiwe et al. provided evidence for S1P as a potential new biomarker during cardiac surgery. They found that serum levels of S1P decreased immediately after surgery and, in patients whose levels failed to reach baseline levels, the ICU stay was prolonged and postoperative inflammation prolonged. A critical limitation of this study was the potential inhibitory of heparin, which may have influenced serum levels during and after surgery. In addition, in a recent small randomized controlled clinical trial, fingolimod, an S1P agonist approved for the treatment of MS, failed to improve overall outcomes in COVID-19 patients but was associated with a lower re-admission rate (5). S1P is an interesting molecule, but its role, whether biomarker or potential drug target in ICU patients, has yet to be determined.

## Outcome predictors

A total of six papers described outcome predictors of ICU populations. The first paper by Choon et al. examined the association between completeness of discharge documentation and subsequent follow-up of acute kidney injury (AKI) survivors who required kidney replacement therapy (KRT) treated at the intensive care unit (ICU) in a retrospective cohort study. The development of AKI and the need for KRT were mentioned in 85 and 82% of critical care discharge letters, respectively. Monitoring kidney function post-discharge was recommended in 51.6% of critical care and 36.3% of hospital discharge summaries. At 3 months, creatinine and urine protein were measured in 88.2 and 11.8% of survivors, respectively. The prevalence of chronic kidney disease stage III or worse increased from 27.2% before hospitalization to 54.9% 1 year after that. These data demonstrate that discharge summaries of patients with AKI who received KRT lacked essential information. Furthermore, renal follow-up was poor in patients with appropriate documentation, suggesting the need for more education and streamlined care pathways. This is especially relevant since failure to record an episode of AKI treated with KRT can have serious implications for patients' future long-term management (6).

The paper by Zhang et al. aimed to explore the clinical features and mortality risk factors of patients with antineutrophil cytoplasmic antibody-associated vasculitis (AAV) requiring ICU treatment. The authors identified that active vasculitis was the most frequent reason for ICU admission, and the leading cause of death was an infection. Acute Physiology and Chronic Health Evaluation II (APACHE II) at admission and respiratory failure were independent risk factors. At the same time, hemoglobin was an independent protective factor of in-ICU mortality for AAV patients admitted to the ICU. The risk prediction model developed in this study may be a helpful tool for clinicians in the early recognition of high-risk patients in this population to apply appropriate management. Because the inconsistent predictive value of biological markers (including hemoglobin) has previously been described, this multivariate model may improve risk prediction in this patient population (7, 8).

The study conducted by Liou et al. aimed to investigate the outcome differences between bystander cardiopulmonary resuscitation (BCPR) and no-BCPR in patients who received Targeted temperature management (TTM) after cardiac arrest. After undertaking a multiple logistic regression analysis, the authors found that BCPR was a significant positive predictor for in-hospital survival. In conclusion, this study demonstrated that BCPR had a favorable survival and neurological impact on spontaneous circulation (ROSC) return in patients receiving TTM after cardiac arrest. While the survival and neurological benefits of BCPR have already been described, this study expands our current knowledge about the outcome benefits of BCPR in patients receiving TTM (9).

The article by Bansal et al. described the development and validation of a multivariate Re-Intubation Summation Calculation (RISC) score for the prediction of respiratory failure after extubation. Predictors of extubation failure included body mass index < 18.5 kg/m^2^, a threshold of Glasgow Coma Scale (GCS) of at least 10 points, mean airway pressure at 1 min of spontaneous breathing trial < 10 cmH_2_O, fluid balance ≥1,500 mL 24 h preceding extubation, and total mechanical ventilation for ≥5 days. Multivariate logistic regression demonstrated that an increase of 1 in the RISC score significantly increased the odds ratio for extubation failure by 1.6-fold. These variables are available in the electronic medical record. The risk prediction model developed in this study may be helpful for clinicians in identifying patients at risk for extubation failure. Since extubation failure is associated with adverse outcomes, including increased hospital mortality, prolonged hospitalization, and increased requirement for tracheotomy, this topic is of great relevance (10, 11).

In the study by Yin et al., a novel critical illness prediction system combining baseline risk factors with dynamic laboratory tests was evaluated in patients with coronavirus disease 2019 (COVID-19). A baseline nomogram model to predict the risk for critical illness at admission consisted of seven variables: age, sequential organ failure assessment (SOFA) score, neutrophil-to-lymphocyte ratio (NLR), D-dimers, lactate dehydrogenase (LDH), international normalized ratio (INR), and pneumonia are interpreted from computed tomography (CT) images. In addition, a linear mixed model (LMM) predicting the occurrence time of critical illness onset during hospitalization based on the dynamic change of seven variables was identified: SOFA score, NLR, C-reactive protein (CRP), glucose, D-dimers, LDH, and blood urea nitrogen (BUN). During the ongoing COVID-19 pandemic, this predictive system could assist in accurately and dynamically predicting critical illness in patients with COVID-19 for appropriate management. This study confirms our current knowledge about risk predictors for severe COVID-19 (12–14).

In the opinion article by Calmels et al., the authors summarize the current knowledge about motor simulation as a plausible non-invasive, safe, easy to implement, and low-cost complementary adjunct among the healthcare delivery provided during the (post-) ICU recovery process. In summary, multidisciplinary healthcare professionals may consider motor simulation as a practical, relevant, and therapeutic option to maximize a patient's return to autonomy.

## Conclusions

In this Research Topic, many research areas from different specialties were covered. While many papers focused on outcome predictors, others investigated the effects of infusions, medications, or analyzed new biomarkers. We are confident that many of the presented articles provide relevant data for critical care specialists across all specialties and settings.

## Author contributions

PK and BT conceived the article and co-wrote the manuscript. All authors contributed to the article and approved the submitted version.

## References

[1] Ely EW Margolin R Francis J May L Truman B Dittus R. Evaluation of delirium in critically ill patients: validation of the Confusion Assessment Method for the Intensive Care Unit (CAM-ICU). Crit Care Med. (2001) 29:1370–9. 10.1097/00003246-200107000-00012 11445689

[2] Marra A Ely EW Pandharipande PP Patel MB. The ABCDEF bundle in critical care. Crit Care Clin. (2017) 33:225–43. 10.1016/j.ccc.2016.12.005 28284292 PMC5351776

[3] Hammond NE Zampieri FG Di TGL Garside T Adigbli D Cavalcanti AB. Balanced crystalloids versus saline in critically Ill adults — A systematic review with meta-analysis. NEJM Evid. (2022) 1:EVIDoa2100010. 10.1056/EVIDoa2100010 38319180

[4] Burg N Salmon JE Hla T. Sphingosine 1-phosphate receptor-targeted therapeutics in rheumatic diseases. Nat Rev Rheumatol. (2022) 18:335–51. 10.1038/s41584-022-00784-6 35508810 PMC9641499

[5] Teymouri S Pourbayram Kaleybar S Hejazian SS Hejazian SM Ansarin K Ardalan M. The effect of fingolimod on patients with moderate to severe COVID-19. Pharmacol Res Perspect. (2023) 11:e01039. 10.1002/prp2.1039 36567519 PMC9791159

[6] Elvey R Howard SJ Martindale A-M Blakeman T. Implementing post-discharge care following acute kidney injury in England: a single-centre qualitative evaluation. BMJ Open. (2020) 10:e036077. 10.1136/bmjopen-2019-036077 32792434 PMC7430404

[7] Ge Y Yang G Yu X Sun B Zhang B Yuan Y. Outcome predictors of biopsy-proven myeloperoxidase-anti-neutrophil cytoplasmic antibody-associated glomerulonephritis. Front. Immunol. (2021) 11:607261. 10.3389/fimmu.2020.607261 33613528 PMC7889806

[8] Wacrenier S Boud'hors C Piccoli G Augusto J-F Brilland B. Commentary: Outcome predictors of biopsy-proven myeloperoxidase-anti-neutrophil cytoplasmic antibody-associated glomerulonephritis. Front Immunol. (2021) 12:691179. 10.3389/fimmu.2021.691179 34149733 PMC8208033

[9] Song J Guo W Lu X Kang X Song Y Gong D. The effect of bystander cardiopulmonary resuscitation on the survival of out-of-hospital cardiac arrests: a systematic review and meta-analysis. Scand J Trauma Resusc Emerg Med. (2018) 26:86. 10.1186/s13049-018-0552-8 30309373 PMC6182861

[10] Epstein SK Ciubotaru RL Wong JB. Effect of failed extubation on the outcome of mechanical ventilation. Chest. (1997) 112:186–92. 10.1378/chest.112.1.186 9228375

[11] Frutos-Vivar F Esteban A Apezteguia C González M Arabi Y Restrepo MI. Outcome of reintubated patients after scheduled extubation. J Crit Care. (2011) 26:502–9. 10.1016/j.jcrc.2010.12.015 21376523

[12] Caricchio R Gallucci M Dass C Zhang X Gallucci S Fleece D. Preliminary predictive criteria for COVID-19 cytokine storm. Ann Rheum Dis. (2021) 80:88–95. 10.1136/annrheumdis-2020-218323 32978237

[13] Tampe D Winkler MS Korsten P Hakroush S Moerer O Tampe B. Correspondence on “Preliminary predictive criteria for COVID-19 cytokine storm”. Ann Rheum Dis. (2021). 10.1136/annrheumdis-2020-219709 33414185

[14] Caricchio R Gallucci M Dass C Zhang X Gallucci S Fleece D. Response to: “Correspondence on “Preliminary predictive criteria for COVID-19 cytokine storm” by Tampe et al. Ann Rheum Dis. (2021). 10.1136/annrheumdis-2020-219720 33414184

